# A *de novo* deletion underlying spinal muscular atrophy: implications for carrier testing and genetic counseling

**DOI:** 10.1093/hmg/ddaf035

**Published:** 2025-03-17

**Authors:** Maria M Zwartkruis, Mirjam S de Pagter, Demi Gommers, Marije Koopmans, Cecile P E Ottenheim, Joris V Kortooms, Mirjan Albring, Martin G Elferink, Renske I Wadman, Fay-Lynn Asselman, Inge Cuppen, W Ludo van der Pol, Marcel R Nelen, Gijs W van Haaften, Ewout J N Groen

**Affiliations:** Department of Neurology and Neurosurgery, UMC Utrecht Brain Center, University Medical Center Utrecht, Heidelberglaan 100, Utrecht 3584 CX, the Netherlands; Department of Genetics, University Medical Center Utrecht, Heidelberglaan 100, Utrecht 3584 CX, the Netherlands; Department of Genetics, University Medical Center Utrecht, Heidelberglaan 100, Utrecht 3584 CX, the Netherlands; Department of Neurology and Neurosurgery, UMC Utrecht Brain Center, University Medical Center Utrecht, Heidelberglaan 100, Utrecht 3584 CX, the Netherlands; Department of Genetics, University Medical Center Utrecht, Heidelberglaan 100, Utrecht 3584 CX, the Netherlands; Department of Genetics, University Medical Center Utrecht, Heidelberglaan 100, Utrecht 3584 CX, the Netherlands; Department of Human Genetics, Amsterdam UMC, University of Amsterdam, Meibergdreef 9, Amsterdam 1105 AZ, the Netherlands; Department of Neurology and Neurosurgery, UMC Utrecht Brain Center, University Medical Center Utrecht, Heidelberglaan 100, Utrecht 3584 CX, the Netherlands; Department of Genetics, University Medical Center Utrecht, Heidelberglaan 100, Utrecht 3584 CX, the Netherlands; Department of Genetics, University Medical Center Utrecht, Heidelberglaan 100, Utrecht 3584 CX, the Netherlands; Department of Neurology and Neurosurgery, UMC Utrecht Brain Center, University Medical Center Utrecht, Heidelberglaan 100, Utrecht 3584 CX, the Netherlands; Department of Neurology and Neurosurgery, UMC Utrecht Brain Center, University Medical Center Utrecht, Heidelberglaan 100, Utrecht 3584 CX, the Netherlands; Department of Neurology and Neurosurgery, UMC Utrecht Brain Center, University Medical Center Utrecht, Heidelberglaan 100, Utrecht 3584 CX, the Netherlands; Department of Neurology and Neurosurgery, UMC Utrecht Brain Center, University Medical Center Utrecht, Heidelberglaan 100, Utrecht 3584 CX, the Netherlands; Department of Genetics, University Medical Center Utrecht, Heidelberglaan 100, Utrecht 3584 CX, the Netherlands; Department of Genetics, University Medical Center Utrecht, Heidelberglaan 100, Utrecht 3584 CX, the Netherlands; Department of Neurology and Neurosurgery, UMC Utrecht Brain Center, University Medical Center Utrecht, Heidelberglaan 100, Utrecht 3584 CX, the Netherlands

**Keywords:** spinal muscular atrophy, long-read sequencing, clinical genetics, carrier screening, survival motor neuron

## Abstract

Spinal muscular atrophy (SMA) is an autosomal recessive disease most commonly caused by homozygous deletion of the *SMN1* gene. Parents of affected children are typically carriers, with a recurrence risk of 25% for future pregnancies. Their close relatives have up to 50% chance of being carriers. Carriers typically possess a single copy of the *SMN1* gene; however, some parents carry two copies of *SMN1*. Current standard diagnostic carrier tests are unable to distinguish between silent carriers with two copies on one chromosome (2 + 0 genotype) and non-carriers (1 + 1 genotype), where a *de novo* deletion occurred. This distinction is crucial for recurrence risk assessment, which highlights the unsolved challenge to carrier testing and genetic counseling. We combined microsatellite marker analysis, *SMN* copy number analysis, Sanger sequencing, long-read sequencing and *de novo* assembly to investigate the cause of the absence of *SMN1* in a pedigree with an SMA patient identified through newborn screening, whose parents each carried two *SMN1* copies. Our analysis revealed that the father is a silent carrier, while *de novo* assembly of the *SMN* locus showed a 1.4 megabase (Mb) *de novo* deletion between mother and child. This deletion encompasses *SMN1* and *SMN2* and represents the first reported nucleotide-level resolved SMA-causing deletion to date. Our findings allowed informed counseling of at-risk relatives and illustrate the complexity of SMA carrier testing and counseling. This case underscores the feasibility of and need for advanced genetic testing for SMA carriership in select cases, to improve genetic counseling practices, risk assessment, and family planning.

## Introduction

Spinal muscular atrophy (SMA) is a severe neuromuscular disorder characterized by progressive muscle weakness and atrophy. The disease is primarily caused by homozygous deletions of the *survival motor neuron 1* (*SMN1*) gene (0 + 0 genotype) [1], with disease severity modulated by the variation in copy number of the highly homologous *SMN2* gene [2–4]. Parents of affected children are typically carriers and couples have a 25% recurrence risk for future pregnancies. Moreover, their close relatives have up to 50% chance of being a carrier. Carriers typically possess one functional copy of *SMN1* (1 + 0 genotype), but some parents have two *SMN1* copies [5, 6]. This implies one of the following mechanisms: 1) the parent is a silent carrier, i.e. the two *SMN1* copies are on the same chromosome (2 + 0 genotype); 2) the parent is not a carrier (1 + 1 genotype), but *de novo* deletion or mutation events occurred in parental gametes; 3) gonadal mosaicism of the parent; or 4) presence of intragenic mutations in *SMN1* (1 + 1^D^ genotype) [7]. Each of these scenarios comes with its own recurrence risk and consequences for family counseling. Current standard of care diagnostic carrier tests are unable to distinguish between these cases, complicating genetic counseling and risk assessment [5, 6]. The advent of newborn screening for SMA is revolutionizing patient care and genetic counseling, offering the potential for early, life-saving interventions. This has made accurate determination of carrier status more critical than ever [8, 9].

The frequency of SMA carriers varies across populations [10]. Similarly, the prevalence of silent carriers and *de novo* events in SMA differs across populations and has been the subject of numerous studies. Table 1 includes an overview of studies performed that included a minimum of 40 parents of SMA patients with homozygous *SMN1* deletions. Silent carriers are estimated to account for 1.0%–5.0% of carriers in most populations (Table 1). While some genetic markers have been described that may be specific to silent carrier alleles [11, 12], their utility is population-specific, with limited predictive power, and thus insufficient for identification of all silent carrier alleles [6, 13–15]. *De novo* events occur in 1.0%–3.4% of parents of SMA patients with homozygous *SMN1* deletion (Table 1). Varying *de novo* deletion sizes have been documented, ranging from a small c.41_42delinsC variant [16] to *de novo* gene conversions [17, 18], to deletions encompassing single exons [19] or multiple genes [20]. These events occur in the context of the complex genomic architecture of the *SMN* locus, which is a region prone to structural variations due to segmental duplications [21, 22]. Despite their clinical significance for recurrence risks, testing for silent carriers or *de novo* events is complicated. This is mostly due to the complex nature of the *SMN* locus and the technical limitations of current standard of care diagnostic SMA testing, which relies heavily on copy number. Relevant family members such as grandparents and/or siblings are essential for haplotype analysis, but their DNA is frequently not available [6]. In addition, carrier detection when screening the general population is complicated by the lack of generalized markers for silent carriership [6]. Therefore, more advanced genetic testing strategies are needed.

**Table 1:** Overview of studies on carriership in parents of SMA patients (only including patients with homozygous *SMN1* deletion).

Publication	Country/region	Number of parents	Number of parents with two copies of *SMN1*	Silent carriers (2 + 0 genotype), n (percentage)	*De novo* structural rearrangement, n (percentage)	Undetermined	Methods used	Evidence
McAndrew *et al.*, 1997 [39]	United States and Canada	79^a^	1/79 (1.3%)^a^	n.a.	n.a.	1 (1.3%)^a^	*SMN1* dosage by competitive quantitative PCR, marker analysis	n.a.
Wirth *et al.*, 1997 [17]	Germany	680	n.a.	n.a.	7 (1.0%), including one *de novo* gene conversion	n.a.	PCR to assess presence of *SMN1* and *NAIP,* marker analysis	Inclusion of siblings and/or grandparents
Chen *et al.*, 1999 [40]	United States	58	4/58 (6.9%)	2 (3.4%)	2 (3.4%)	0 (0.0%)	*SMN1* dosage by competitive quantitative PCR, marker analysis	Inclusion of siblings and/or grandparents
Scheffer *et al.*, 2000 [41]	The Netherlands	63	2/63 (3.2%)	n.a.	n.a.	2 (3.2%)	*SMN1* dosage by competitive quantitative PCR	n.a.
Mailman *et al.*, 2001 [42]	North America	100	4/100 (4.0%)	1 (1.0%)	1 (1.0%)	2 (2.0%)	Mouse/human somatic cell hybridization, *SMN1* dosage by competitive quantitative PCR, marker analysis, FISH	Analysis of single chromosomes
Ogino *et al.*, 2002 [43]	United States North East region	102	6/102 (5.9%)	4 (3.9%)	2 (2.0%)	0 (0.0%)	*SMN1* dosage by competitive quantitative PCR, marker analysis	Inclusion of siblings and/or grandparents
Cusin *et al.*, 2003 [18]	France	202	9/202 (4.5%)	6 (3.0%)	3 (1.5%), including one *de novo* gene conversion	0 (0.0%)	*SMN1* dosage by competitive PCR, marker analysis	Inclusion of siblings and/or grandparents
Smith *et al.*, 2007 [44]	Australia (Victoria)	117	7/117 (6.0%)	2 (1.7%)	2 (1.7%)	3 (2.6%)	*SMN1* dosage by competitive quantitative PCR, marker analysis	Inclusion of siblings and/or grandparents
Sheng-Yuan *et al.*, 2010 [45]	China	40	2/40 (5.0%)	2 (5.0%)	0 (0.0%)	0 (0.0%)	Modified denaturing high-performance liquid chromatography (DHPLC) based quantitative assay	2 + 0 carrier state is inferred from copy number of parents only
Alías *et al.*, 2014 [46]	Spain	488	21/488 (4.3%), 1/488 (0.2%) had three *SMN1*	15 (3.1%)	5 (1.0%)	1 (0.2%)	Quantitative real-time PCR, additional MLPA in carriers with 2 copies *SMN1*, marker analysis	Inclusion of siblings and/or grandparents
Ar Rochmah *et al.*, 2017 [47]	Japan	63	2/63 (3.2%)	1 (1.6%)	0 (0.0%)	1 (1.6%)	real-time PCR, MLPA	2 + 0 carrier state is inferred from *(pseudo)NAIP* copy number in trio only
Davidson *et al.*, 2023 [48]	Australia (NSW)	116	8/116 (6.9%), 1/116 (0.9%) had zero *SMN1*	4 (3.4%) probable silent carriers	0 (0.0%)	4 (3.4%)	MLPA	Inclusion of siblings in two cases
Bai *et al.*, 2024 [16]	China	64^b^	15/64 (23.4%)^b^	15 (23.4%)^b^	0 (0.0%)	0 (0.0%)	MLPA, long-range PCR followed by PacBio HiFi sequencing	Inheritance pattern of *SMN1/2* copies in pedigrees determined by sequencing, additional relatives were included in some cases.

^a^Carrier parents were selected based on segregation analysis of marker Ag1-CA, so this percentage might not be representative for the population.

^b^Patients and parents were retrospectively selected, so this percentage might not be representative for the population.

Current practices in genetic counseling for SMA vary in their approach to parental testing and risk assessment [23, 24]. The frequency of silent carriership and the counseling procedures for parents with two *SMN1* copies are areas that require further investigation. In this study, we illustrate this through a case of a child with SMA with homozygous deletion of *SMN1* and three *SMN2* copies, whose parents both initially appeared to carry two intact *SMN1* genes. Through a comprehensive approach combining pedigree marker and single nucleotide variant (SNV) analysis, *SMN* copy number analysis, long-read sequencing and *de novo* assembly, we demonstrate that the father is a silent carrier and that a *de novo* deletion of 1.4 megabases (Mb), encompassing both *SMN* genes, occurred on the maternal chromosome. This highlights the importance of advanced genetic testing in complex SMA cases and illustrates a strategy for differentiating between silent carriers and *de novo* events. Our findings have significant implications for genetic counseling, recurrence risk assessment, and family planning and show that refined carrier testing strategies can be successful.

## Results

### Case presentation

The proband, the first child of non-consanguineous parents, was born after an uneventful pregnancy (duration of 39 weeks and 6 days; Apgar scores 9/9/8 after 1, 5 and 10 minutes; weight at birth 3510 grams). She was treated with continuous positive airway pressure (CPAP) and oxygen for 24 h due to wet lung and after improvement she was discharged. On day 10 after birth, parents received the news that newborn screening showed a homozygous deletion of the *SMN1* gene. At the time of first consultation on day 11, there were no signs of weakness or respiratory or feeding abnormalities. Neurological examination was normal and all reflexes could be elicited. CHOP-INTEND score [25] was 55 out of a total of 64 (normal). Genetic testing confirmed the absence of the *SMN1* gene and the presence of three copies of the *SMN2* gene. Treatment with onasemnogene abeparvovec on day 31 was without complications and the proband showed normal gross motor development afterwards. She was able to sit independently at the age of 7 months, stand and cruise at the age of 9 months and walked independently shortly before her first birthday.

### Pedigree analysis

Carrier testing of the parents was performed to assess recurrence risk for the parents and other relatives. *SMN1* and *SMN2* copy number analysis showed that both parents carried two copies of the *SMN1* gene. Short tandem repeat (STR) profiling analysis confirmed the biological relationship of the child and parents, ruled out a potential sample swap and determined that the parents were non-consanguineous. To assess the possibility of silent carriership, *SMN1/2* copy number and marker analysis was performed in the grandparents and other relevant family members (Fig. S1). The paternal grandfather I-1 carried an *SMN1* deletion allele, and the paternal grandmother I-2 carried an *SMN1* duplication allele, making the father II-1 a likely silent carrier of SMA (2 + 0 genotype). DNA from the maternal grandfather (I-4) was unavailable. Therefore, the maternal great-uncle (I-5) and his family were included for further analysis. Individual I-5 had two *SMN1* gene copies and was found to have the same chromosome 5 genotype as maternal grandfather (I-4). The absence of duplications or deletions in the mother’s blood relatives (II-3, II-4, I-3, I-5) suggests that a *de novo* event is more probable than silent carriership. However, exact inheritance patterns could not be determined without additional genetic information.

### Long-read sequencing and *SMN1/2* haplotype analysis

**Figure 1:**
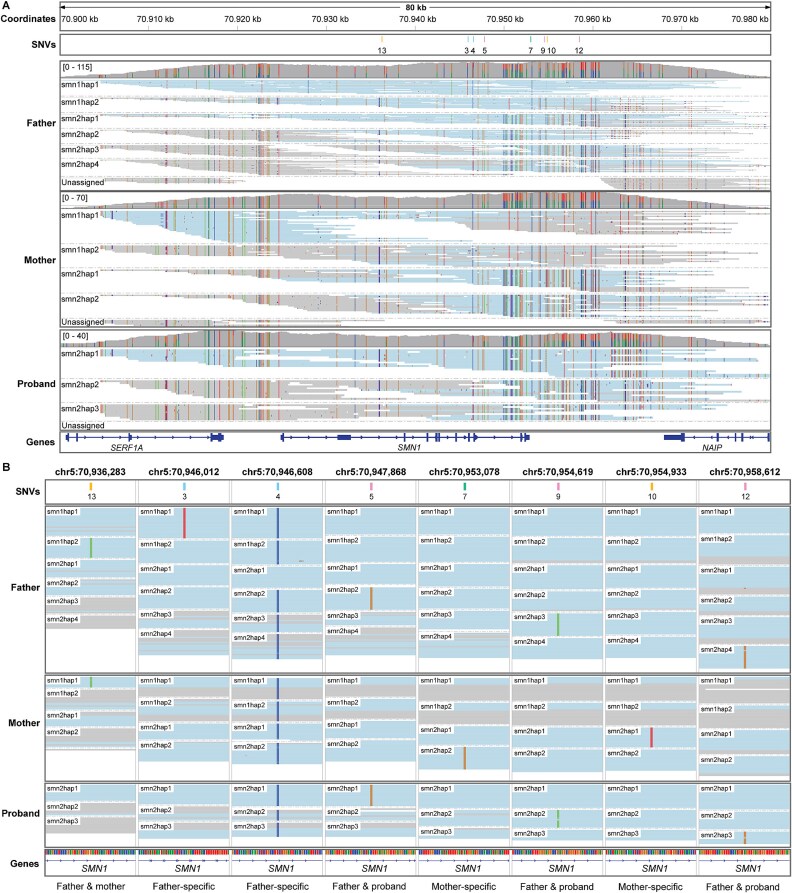
*SMN1/2* haplotype analysis of the proband, father and mother showing that all *SMN2* copies of the proband were inherited from the father. (A) IGV snapshot of PacBio HiFi sequencing reads, aligned to *SMN1* on the GRCh38 reference genome and phased into *SMN1/2* haplotypes by Paraphase [11]. Father has haplotypes of haplogroups S1–2, S1–1, S2–2, S2–1, S2–1, S2–1, mother S1–1, S1–1, S2–1, S2–1 and proband S2–1, S2–1, S2–1 (listed as shown from top to bottom). Since these haplogroups are largely similar between father and mother, investigation of additional SNVs was needed to discern haplotypes from each other. (B) IGV snapshot of data in (A), zoomed in on SNVs that mark specific *SMN1/2* haplotypes. SNVs are colored according to the *SMN1/2* haplotypes that they mark in Fig. 2. SNV 5, 9 and 12 mark the three *SMN2* haplotypes that were inherited from the father by the proband. SNV 3, 4 (reference allele) and 13 mark the *SMN* haplotypes on the father’s allele that was not passed on to the proband. SNV 7, 10 and 13 discern the different *SMN* haplotypes in the mother from each other, which can be used to determine the inheritance pattern in the maternal side of the pedigree (*SMN1*hap2 could not be uniquely identified by a single SNV in the mother).

To further investigate inheritance patterns of *SMN1* and *SMN2*, PacBio HiFi sequencing was performed on the trio to a read depth of 25-37x and read length N50 of 18–20 kilobases (kb) per sample. Paraphase analysis for *SMN1/2* [11] was performed on the trio. *SMN1/2* copy numbers determined with Paraphase were concordant with Amplidex and multiplex ligation-dependent probe amplification (MLPA) results. Father carried haplotypes belonging to haplogroups S1–2, S1–1, S2–2, and S2–1 (three copies); mother S1–1 (two copies) and S2–1 (two copies); and proband S2–1 (three copies, Fig. 1A). Co-segregation of haplogroups S1–8 and S1-9d, a predictor of silent carriership (88.5% in African individuals) [11] was not found in this family. Two tightly linked variants—g.27134 T > G in intron 7 (rs143838139) and g.27706_27707delAT in exon 8 (rs200800214)—are strongly associated with *SMN1* duplication alleles in the Ashkenazi Jewish population [12] and indicate 1%–8% chance of silent carriership when *SMN1* copy number is two in other populations [6, 13–15]. These variants were not detected in this family. However, by analyzing haplotype-specific SNVs, we determined that all *SMN2* haplotypes of the proband were inherited from the father (haplotypes *SMN2*hap2, *SMN2*hap3, *SMN2*hap4). Therefore, the remaining haplotypes, including two *SMN1* copies, are located on the other allele, confirming the silent carrier status of the father (2 + 0 genotype, Fig. 1B).

### Additional SNV analysis

**Figure 2:**
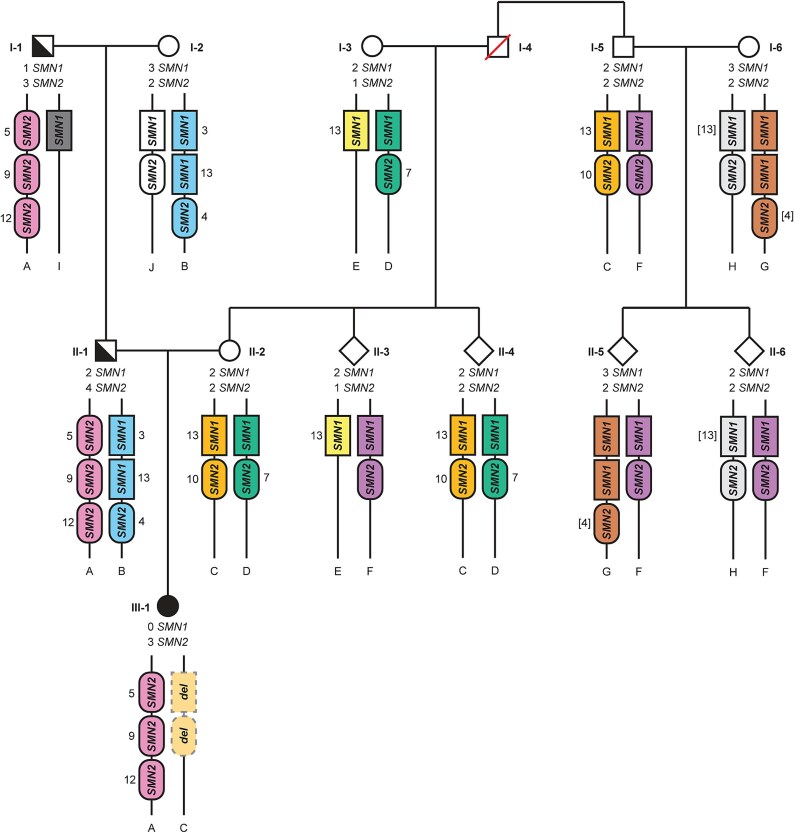
Pedigree analysis shows that the father of the proband is a silent carrier of SMA, and that the mother is not a carrier of SMA. Filled symbols represent individuals with SMA, half-filled symbols represent SMA carriers. *SMN1/2* copy number is indicated per individual. Alleles are colored and marked by letters A-J. SNVs that mark specific *SMN* copies (determined by PacBio HiFi sequencing (Fig. 1) and Sanger sequencing (Table S1)) are shown by numbers next to the *SMN* gene copies. If a SNV number is between brackets, it is not certain on which *SMN1/2* copy of the allele it is located.

To determine the inheritance pattern in the rest of the pedigree, we performed Sanger sequencing on the SNVs determined by PacBio HiFi sequencing (Table S1). We confirmed the expected inheritance pattern on the paternal side of the pedigree based on six haplotype-specific SNVs (Fig. 2). In the mother, SNV 10 and 13 co-segregated on one allele, and SNV 7 was located on the other allele. Therefore, one allele must contain *SMN1* and *SMN2* and the other allele at least one copy of *SMN2*. *SMN1*hap2 of the mother could not be identified by a unique SNV, but copy number and haplotype analysis of individual I-5 and his nuclear family (I-6, II-5, II-6) revealed that this *SMN1* copy was located on allele D (Fig. 2): allele F must contain one *SMN1* copy, and therefore allele C cannot contain two *SMN1* copies. Thus, the mother has a 1 + 1 *SMN1* genotype, and therefore, a *de novo* deletion of a region containing *SMN1* and *SMN2* between mother and proband is the only possible explanation.

### *De novo* assembly to determine size of *de novo* deletion

**Figure 3:**
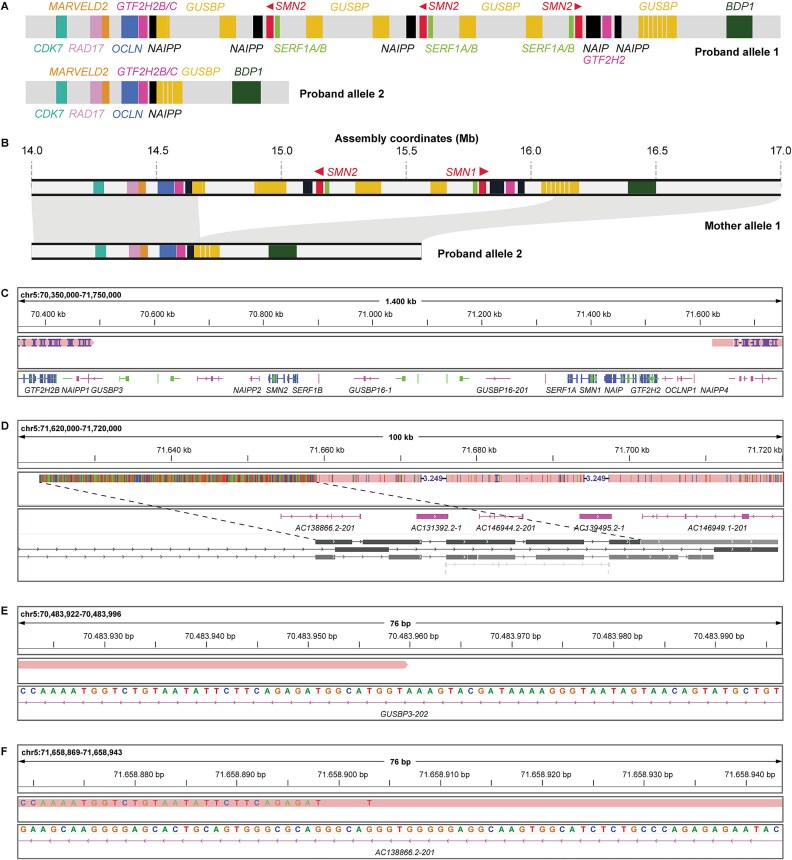
*De novo* assembly of the *SMN* locus indicates a 1.4 Mb maternal *de novo* deletion that includes *SMN1* and *SMN2.* (A) Structure of the *SMN* locus in the paternal allele 1 (top) and maternal allele 2 (bottom) of the proband. The paternal allele contains three *SMN2* copies and the maternal allele contains zero *SMN* copies, as expected by haplotype analysis in Fig. 1 and 2. Gene names are indicated in the figure. (B) Synteny plot of alignment between allele 1 of the mother and (maternal) allele 2 of the proband. Genes have the same color as in (A). A 1.4 Mb fragment containing both *SMN1* and *SMN2* was deleted between the mother and the proband. This deletion was likely mediated by repetitive *GUSBP* sequences in the *SMN* locus of the mother. The shown coordinates are relative to the start of the resolved allele 1 of the mother (roughly corresponding to GRCh38 chr5:55000000). (C) The assembly of allele 2 of the proband mapped to the T2T-CHM13 reference genome, visualizing both sides of the *de novo* deletion breakpoint. (D) Visualization of the right side of the *de novo* deletion breakpoint; the soft-clipped sequence was aligned to the reference sequence using BLAT, results are shown in grey below the gene annotations. The soft-clipped sequence is likely a duplication of the sequence to its right (see dashed lines). The transcript *AC138866.2–201* corresponds to *GUSBP9* according to the NCBI RefSeq annotation in the UCSC genome browser. (E) Single-nucleotide view of the left side of the *de novo* deletion breakpoint. (F) Single-nucleotide view of the right side of the *de novo* deletion breakpoint. The soft-clipped sequence resembles the reference sequence above (E).

To determine the size of the *de novo* deletion, we performed additional targeted Oxford Nanopore Technologies (ONT) long-read sequencing of the father, mother and proband to an on-target read depth of 11–23x and read length N50 of 36-43 kb per sample. We combined PacBio HiFi and ONT sequencing data to perform *de novo* assembly of the 30 Mb region surrounding the *SMN* locus. Unphased assembly graphs of the trio, focusing on the *SMN* locus, are shown in Fig. S2–S4. For the proband, we used the available PacBio HiFi data of the parents to perform haplotype phasing of the assembly. As expected, the paternal allele contained three *SMN2* copies, and the maternal allele contained zero *SMN1/2* copies (Fig. 3A). One allele of the mother could be resolved completely (Fig. 3B) and has an *SMN* locus more similar to the telomere-to-telomere CHM13 (T2T-CHM13) reference genome than to the GRCh38 reference genome (Fig. S5A and B). To determine the *de novo* deletion size and visualize breakpoints, we aligned the maternal allele of the proband to allele 1 of the mother (Fig. 3B). This revealed the *de novo* deletion is 1 446 974 base pairs (bp) (1.4 Mb) in size relative to allele 1 of the mother, corresponding to a 1 174 947 bp (1.2 Mb) fragment (chr5:70483959–71658906) in the T2T-CHM13 reference genome (Fig. 3C–F, Fig. S5C and D). Breakpoints were located within repetitive *glucuronidase beta pseudogene (GUSBP)* sequences. The *GUSBP* pseudogenes are part of the core duplicon that expanded specifically in the human lineage and is present in two tandem and 10 dispersed copies on chromosome 5 and one copy on chromosome 6, that is presumed to be the ancestral copy [26]. We found that numerous repetitive elements resembling *GUSBP* pseudogenes are located throughout the *SMN* locus of the mother, in a higher copy number than in the reference genomes (as visible by multiple small diagonal lines above each other in the dot plot, Fig. S5A and B). Since these repetitive *GUSBP* sequences are also located around the DNA fragment that is missing in the proband, the deletion was likely mediated by these sequences (Fig. 3B, Fig. S5C and D). Indeed, the left breakpoint was located in *GUSBP3* and the right breakpoint in *GUSBP9* in the T2T-CHM13 reference genome (Fig. 3D–F). By confirming the occurrence of a *de novo* deletion between the mother and the proband, we demonstrate that there are no SMA carriers within the maternal side of the pedigree. (Fig. 2).

## Discussion

In this study, we report a rare example of a child affected with SMA born from non-consanguineous parents, who both carry two *SMN1* copies. We combined conventional genetic methods including microsatellite marker analysis, MLPA and Sanger sequencing with innovative long-read sequencing methods to confirm the silent carrier status of the father and to show a *de novo* deletion event of 1.4 Mb including *SMN1* and *SMN2* between the mother and proband. Through this, we determined that no SMA carriers were present in the maternal branch of the pedigree. The family was counseled to have a recurrence risk of less than 1%. This study demonstrates the importance of additional testing to distinguish silent carriership from *de novo* events for genetic counseling in families of SMA patients.

We are the first to confirm a large *de novo* SMA-causing deletion at the nucleotide level using long-read sequencing technologies. Several studies have confirmed *de novo* deletions by ruling out silent carriership through investigation of grandparents and/or (haploidentical) siblings but could not determine the size of deletions due to methodological constraints (Table 1). One previous study reported a 1.3 Mb *de novo* deletion, which is similar in size to the deletion reported in our study. However, as it was based on single nucleotide polymorphism (SNP) array methodology—showing reduction of total *SMN1/2* copy number in the proband—the presented evidence should be regarded as indirect as it cannot exclude the possibility of silent carriership in both parents [27]. The *de novo* deletion in our study seems to be mediated by repetitive sequences in a *GUSBP* pseudogene. The resolved allele 1 of the mother appears to contain more repeat sequences (multiple small diagonal lines above each other in Fig. S5A and B) compared to both the GRCh38 and the T2T-CHM13 reference genome. This could potentially make the genotype of the mother more prone to rearrangements. However, additional sequencing data of the maternal grandparents is necessary to improve phasing of the assembly of the mother into two haplotypes and confirm this genotype. The size and structure of the *SMN* locus varies greatly between individuals [21, 28]. Investigating the genotypes from which SMA-causing *de novo* rearrangements arise could reveal whether specific configurations of the *SMN* locus, such as those with more repetitive sequences, are more susceptible to such rearrangements. This would allow for more accurate genetic counseling concerning recurrence risks in case of *de novo* events.

Silent carriership can currently be determined by detecting a duplication and deletion allele in grandparents and or siblings, as in our study, or when single chromosomes are studied, for example by single sperm analysis or somatic cell hybridization. Some genetic markers (see Results for more detailed description) predict silent carrier status [11, 12], but their utility is population-specific and thus insufficient for identification of all silent carrier alleles [6, 13–15]. The comprehensive analysis of SMA (CASMA) method, based on long-range-PCR followed by PacBio HiFi sequencing [29], can detect silent carriers when performed on a trio [30, 31]. In addition, a set of 31 SNVs downstream of *SMN1* could be used to predict 50% of *SMN1* duplication alleles, increasing carrier detection from 91% to 98% in the Chinese population [29]. Similar downstream SNVs were marked as the ‘c’ haplotype in another study, that was present in 28 (96.6%) out of 29 alleles with two copies of *SMN1* and zero copies of *SMN2*, but not in any of the seven alleles with two copies *SMN1* retaining *SMN2*. This suggests that the ‘c’ haplotype can be used for detection two-copy *SMN1* alleles that arose from gene conversion, but not from duplication [11].The 2 + 0 genotype of the father in our study likely arose from duplication, since the duplication allele retains *SMN2* and no ‘c’ haplotype was detected. In addition, our study shows that silent carriership can be confirmed through long-read sequencing of the person at risk to be a silent carrier, and Sanger sequencing of haplotype-specific SNVs in their parents. However, *SMN1* sequence analysis of confirmed silent carriers from different populations is needed to discover more accessible markers of silent carriership and to increase silent carrier detection in carrier screening of the general population.

Distinguishing between silent carriers and *de novo* rearrangements is not only key for recurrence risk calculations, but also for accurate counseling of family members. Current practice for parents that are found to carry two copies of *SMN1* is to 1) confirm parental status and exclude sample swaps; 2) to determine *SMN1* copy number in the grandparents, where silent carriership is likely if one grandparent has a duplication allele and the other grandparent has a deletion allele; and 3) to perform microsatellite marker analysis and *SMN1* and *SMN2* copy number analysis of additional family members. This allows for the distinction between the different alleles someone has inherited and enables increased accuracy of genetic counseling for family members. If the above-mentioned methods do not provide a conclusive answer, more advanced methods such as long-read sequencing are required. As in our study, PacBio HiFi sequencing can be used to determine *SMN1/2* haplotype-specific SNVs due to its high accuracy and the availability of the Paraphase tool [11]. For high quality *de novo* assembly of complex loci such as the *SMN* locus, both highly accurate PacBio HiFi and ultralong ONT sequencing reads are generally required to span segmental duplications and other repetitive sequences [28]. However, these methods are not commonly available in routine diagnostics, due to their high cost and complexity of analysis. Therefore, detailed carriership studies are required to identify markers of silent carriership that are applicable in routine screening and diagnostics. Our current study provides an outline of the genetic approaches currently required to provide definitive answers around silent carrier status and the occurrence of *de novo* deletion events in what remains one of the most complex loci in the human genome.

In conclusion, we report a rare example of an SMA patient of whom the father is a silent carrier, while a *de novo* deletion of *SMN1* and *SMN2* occurred on the maternal allele. We determined the exact size and location of the deletion through *de novo* assembly of the *SMN* locus. Through this, we show that SMA carriership is not present on the maternal side of the pedigree, allowing informed genetic counseling. Our study demonstrates the importance of additional, extensive genetic testing to distinguish silent carriership from *de novo* events for genetic counseling in families of SMA patients.

## Materials and methods

### Subjects

One SMA patient, two parents and nine other relatives from one pedigree were included in this study. Written informed consent was obtained from the parents for themselves and their child as part of study protocol (09307/NL29692.041.09), that was approved by the Medical Ethical Committee of the University Medical Center Utrecht and was registered in the Dutch registry for clinical studies and trials (https://www.ccmo.nl/).

### DNA extraction

For diagnostic testing and Sanger sequencing, DNA was extracted from EDTA whole blood with the Bl chemagic DNA Blood 4 k kit (Revvity, CMG-1074). For long-read sequencing, high molecular weight (HMW) DNA was extracted from frozen EDTA whole blood of the SMA patient and the parents using the Monarch® HMW DNA Extraction Kit for Cells & Blood (New England Biolabs (NEB), T3050L) as per the manufacturer’s protocol with lysis agitation at 1400 rpm. DNA concentration was measured using the Qubit™ dsDNA Quantification Broad Range Assay (Invitrogen, Q32853) and purity was determined on a spectrophotometer (Nanodrop 2000, Thermo Scientific).

### STR genotyping

STR genotyping was performed using the AmpFLSTR Identifiler Plus PCR Amplification kit (Applied Biosystems, 4 427 368). PCR was performed in a 15 μl reaction consisting of 2 μl genomic DNA (0.5 ng/μl) and 13 μl reaction mix consisting of 6 μl AmpFLSTR Identifiler Plus Master Mix, 3 μl Identifiler Plus Primer Set and 4 μl low TE buffer, according to the following protocol: 11 minutes at 95°C, followed by 28 cycles of 20 seconds at 94°C and 3 minutes at 59°C, and final incubation for 10 minutes at 60°C. 1.3 μl of 30x diluted PCR product was mixed with 8.82 μl of Hi-Di™ Formamide (Applied Biosystems) and 0.18 μl of GeneScan™ 10x diluted LIZ500™ dye size standard (Applied Biosystems). Analysis was performed on a 3730 Genetic Analyzer (Applied Biosystems) using a 36 cm capillary filled with POP-7™ polymer (Applied Biosystems). Data analysis was performed using GeneMarker software version 2.7.0 (SoftGenetics LLC).

### Microsatellite marker analysis

Analysis of eight previously published STR markers located around the SMA locus was performed: D5S1408, D5S1417, D5S610, D5S629 and D5S637, D5S465, D5S681 and D5S1501 [32, 33]. PCR was performed in a 10 μl reaction consisting of 1 μl genomic DNA (~50 ng/μl), 8.42 μl reaction mix (consisting of 1 μl PCR buffer II (Applied Biosystems), 1 μl MgCl2 (Applied Biosystems), 0.1 μl dNTPs (25 mM) and 6.32 μl PCR-grade water), 0.25 μl forward primer (25 μM), 0.25 μl reverse primer (25 μM) and 0.08 μl AmpliTaq Gold DNA polymerase (Applied Biosystems). Thermal cycling was performed with 10 minutes at 95°C, followed by 33 cycles of 30 seconds at 95°C, 30 seconds at 55°C, and 30 seconds at 72°C, and a final incubation for 30 minutes at 72°C. 1.3 μl of 10x diluted PCR product was mixed with 8.82 μl of Hi-Di™ Formamide and 0.18 μl of GeneScan™ 500 ROX™ dye size standard (Applied Biosystems). Analysis was performed on a 3730 Genetic Analyzer using a 36 cm capillary filled with POP-7™ polymer. Data analysis was performed using GeneMarker software version 2.7.0.

### *SMN1/2* copy number analysis

*SMN1* and *SMN2* copy number analysis was performed using the AmplideX SMA Plus Kit (Asuragen, A00056) according to the manufacturers’ instructions. For every test performed, three in-house healthy individuals with two *SMN1* copies and two *SMN2* copies were used as negative controls, and two SMA patients were used as positive controls; one with three *SMN2* copies and one with four *SMN2* copies. 1 μl of undiluted PCR product was mixed with 8 μl of Hi-Di™ Formamide and 1 μl of GeneScan™ 1000 ROX™ dye size standard (Applied Biosystems). Analysis was performed on a 3730 Genetic Analyzer using a 36 cm capillary filled with POP-7™ polymer. Data analysis was performed using GeneMarker software version 2.7.0. In addition, *SMN1* and *SMN2* copy number was determined using MLPA (MRC Holland, SALSA MLPA Probemix P021 SMA Version B1) according to the manufacturer’s protocol (https://www.mrcholland.com/).

### Long-read sequencing

Two ONT PromethION sequencing runs were performed. Per sequencing run, three library preps with 1.3 μg HMW DNA each were made with the ligation sequencing kit (ONT, SQK-NBD114–96). The library preps were sequenced on a FLO-PRO114M flow cell on a PromethION 2 Solo (ONT) connected to a compute unit (ONT GridION) with MinKNOW v23.07.12–24.02.16 and FAST basecalling for 72 h, with a nuclease flush and reloading a new library prep every 24 h. Adaptive sampling to enrich for the *SMN* locus and 30 Mb surrounding sequence was performed as described previously [34]. For PacBio HiFi whole-genome sequencing, samples were processed following the manufacturer’s instructions (Pacific Biosciences (PacBio)). 7–8 μg HMW DNA was sheared on a Megaruptor 3 (Diagenode) to a target size of 15–18 kb. Libraries were prepared with SMRTbell prep kit 3.0 (PacBio) and size-selected for fragments larger than 10 kb on the BluePippin (Sage Science). Final libraries were sequenced for 24 h on the Revio system with 1 SMRT Cell per sample. HiFi reads were generated with CCS 12.0.0.177059.

### Sanger sequencing

For Sanger sequencing, primers and reaction-specific conditions are listed in Table S2. PCRs for SNVs 5, 7 and 13 were performed as follows: each 10 μl PCR reaction contained 5.8 μl PCR-grade water, 1.0 μl 10x NH4 reaction buffer, 0.3 μl 50 mM MgCl_2_ solution, 0.2 μl dNTP Mix (10 mM each), 2.0 μl template gDNA (5 ng/μl), 0.6 μl primer mix (5 μM each) and 0.1 μl BioTaq polymerase (5 U/μl, GC Biotech, BIO-21060). The PCR was run using a Biorad T100 thermocycler (#1861096) with the following protocol: 95°C for 2 minutes; 30 cycles of 95°C for 30 seconds, annealing at variable temperatures for 30 seconds (ramp rate 0.5°C/s), followed by 72°C for 30 seconds (ramp rate 1.1°C/s); and a final elongation at 72°C for 5 minutes. PCRs for SNVs 3, 4, 9, 10 and 12 were performed as follows: each 10 μl PCR reaction contained 2.4 μl PCR-grade water, 2.0 μl template gDNA (5 ng/ul), 0.6 μl primer mix (5 μM each) and 5.0 μl KAPA HiFi HotStart Uracil+ReadyMix (2x) (Roche, 07959052001). The PCR was run using a Biorad T100 thermocycler (#1861096) with the following protocol: 95°C for 3 minutes; 30 cycles of 98°C for 20 seconds, annealing at variable temperatures for 15 seconds (ramp rate 0.5°C/s), followed by elongation at 72°C for 15 seconds (ramp rate 1.1°C/s); and a final elongation at 72°C for 1 minute. For SNV 12, elongation was performed for 2 minutes and 45 seconds per cycle, and the final elongation was 3 minutes and 45 seconds. Sanger sequencing was outsourced to Macrogen. Analysis of Sanger sequencing results was performed in SnapGene v5.1.5. A variant was called if a peak of any size other than the reference allele was present, and the surrounding peaks did not contain any noise.

### Paraphase

PacBio HiFi reads were mapped to GRCh38 with minimap2 v2.26 with options -y -ax map-hifi. Paraphase v2.2.3 [11] was run with option -g smn1. The resulting BAM files were viewed in IGV v2.18.2 with the ‘hide small indels’ (below 5 bp) option. Haplotype-specific SNVs were detected by visual inspection in IGV.

### *De novo* assembly

For each individual, previously mapped PacBio HiFi data was filtered to retain only primary mapped reads within a 30 Mb region surrounding the *SMN* locus (chr5:55000000-85000000) using samtools view v1.17. ONT reads were aligned to the GRCh38 reference genome using minimap2 v2.26 with options -y -ax map-ont, and the resulting BAM files were filtered to retain only primary mapped reads within a 30 Mb region surrounding the *SMN* locus (chr5:55000000-85000000) using samtools view v1.17. BAM files were converted to FASTQ with samtools fastq v1.17 with options -T RG, MM, ML. For each parent, k-mer counts were generated from filtered PacBio HiFi data of each parent using yak count (v0.1) with parameters -k31 and -b37. *De novo* assembly of the parents was performed with hifiasm v0.19.5 [35] with default settings for the father and -D 6 -k 30 for the mother, using both filtered PacBio HiFi and ONT FASTQ files as input. No sequencing data of the grandparents or Hi-C data of the parents was available to aid haplotype phasing, therefore the phased alleles of the parents might contain switch errors. *De novo* assembly of the proband was performed including yak k-mer counts of the parents for trio-binning to aid haplotype phasing. To resolve both haplotypes of the proband, hifiasm settings were optimized; the paternal allele was resolved with default settings and the maternal allele was resolved with settings -D 7 -k 28; the unphased assembly with settings -D 7 -k 28 was shown. The unphased and phased assembly graphs and the T2T-CHM13 and GRCh38 reference genome were visualized with Bandage v0.8.1 [36]. Sequences of the genes within the *SMN* locus were extracted from the T2T-CHM13 reference genome with bedtools v2.30.0; in case of multiple homologous genes or pseudogenes, only one gene was included (*OCLN*: chr5:70317932-70383882; *SERF1A/B*: chr5:71357459-71375322; *SMN1/2*: chr5:71381875-71409804; *NAIP*: chr5:71424931-71482091; *GTF2H2*: chr5:71489386-71524303; *CDK7–201*: chr5:70060645-70103173; *RAD17–207*: chr5:70195082-70240793; *MARVELD2–204*: chr5:70241119-70269905; *BDP1–201*: chr5:71936967-72049156; *MAP1B-201*: chr5:72588652-72690733; *GUSBP16–201*: chr5:71208722-71252673). These sequences were blasted against the assembly graphs using buildblastdb (The National Center for Biotechnology Information) within the Bandage tool, with a minimum percent identity of 90%, alignment length of 1 kb and 10% query coverage to identify the location of the genes on the assemblies. The synteny plot was created with plotsr v1.5.5 (in python v3.11) following the standard pipeline [37]: the resolved allele 1 of the mother was mapped against the maternal allele 2 of the proband with minimap2 v2.26 with settings -ax asm5 –eqx, followed by sorting and indexing via samtools view and index (v1.17). SyRI v1.7.0 was executed on the BAM file with the standard parameters and the output was visualized with plotsr with standard parameters. To generate dot plots, two phased assembly graphs in GFA format were converted to FASTA files for nucmer alignment (MUMmer-4.0, default settings [38]) against each other and the *SMN* locus genes (same as above). Delta-filter was applied to retain alignments with at least 90% identity and a minimum length of 1 kb. Show-coords was used to generate overlapping regions, which were visualized in R v4.4.0 as a dotplot using geom_segment() within the ggplot2 package; the gggenes package was used to determine the location of the genes on the reference (x-axis in dot plots), which was overlaid with the previously mentioned Bandage visualizations. Deletion size and breakpoints were determined from the nucmer show-coords output. The FASTA file of the deletion allele of the proband was split at the deletion breakpoint and mapped to the T2T-CHM13 reference genome using hapdiff v0.8; results were visualized in IGV.

## Supplementary Material

Zwartkruis_et_al_supplementary_ddaf035

Supplementary_table_2_Sanger_amplicon_overview_ddaf035

## Data Availability

The datasets used and/or analyzed during the current study are available from the corresponding author on reasonable request but are not publicly available due to privacy restrictions.
