# Supplementary material for: A de novo deletion underlying spinal muscular atrophy: implications for carrier testing and genetic counseling
Source: Hum Mol Genet. 2025 Mar 17;34(10):894–904. doi: 10.1093/hmg/ddaf035 (PMC12056310; doi:10.1093/hmg/ddaf035)
Supplement: Zwartkruis_et_al_supplementary_ddaf035 [file zwartkruis_et_al_supplementary_ddaf035.pdf]

Supplementary figures and tables

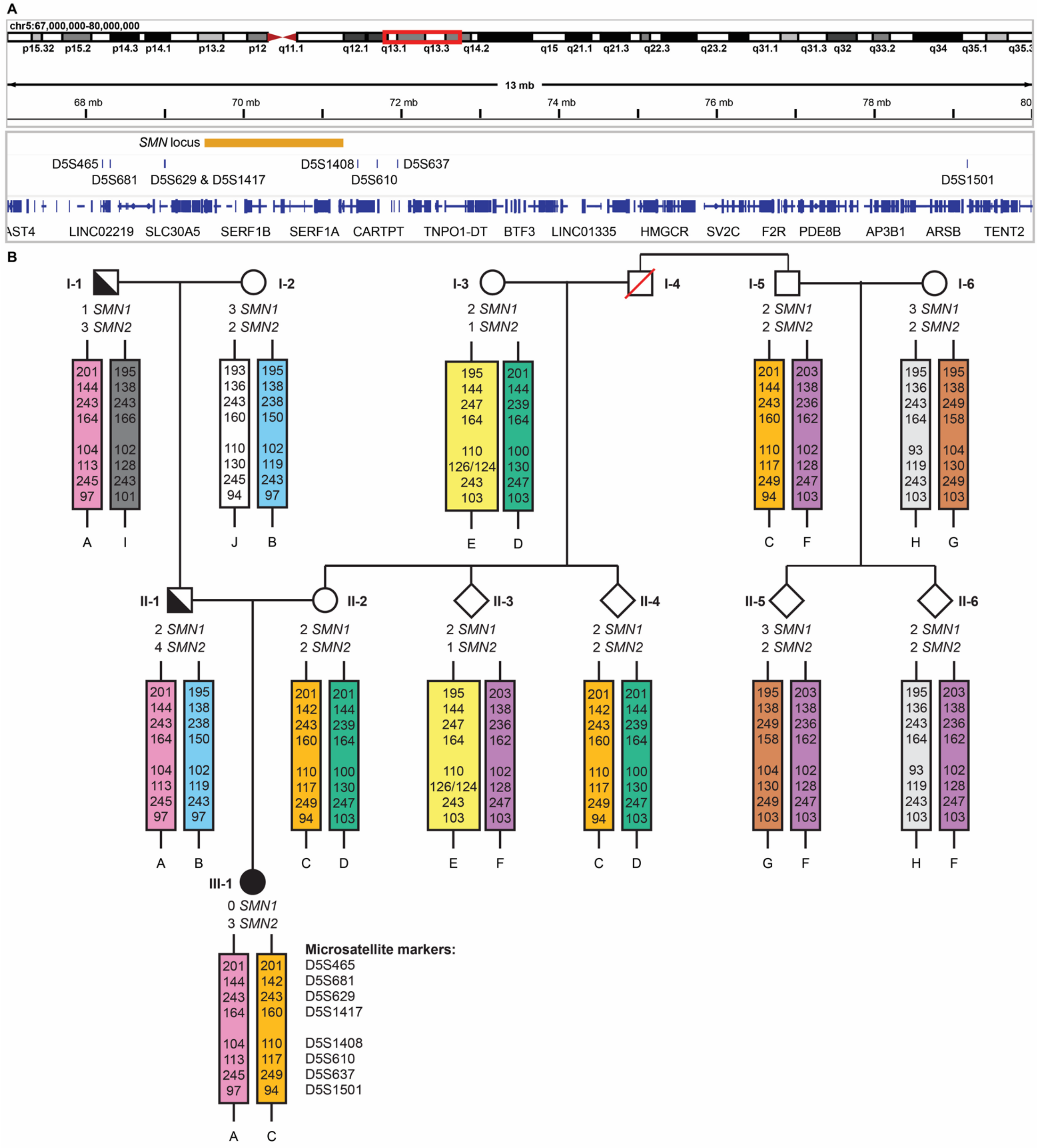

Supplementary figure 1: Haplotype analysis with microsatellite markers surrounding the *SMN* locus.
(A) IGV snapshot of microsatellite marker locations relative to the *SMN* locus on GRCh38.
(B) Haplotype segregation analysis within the pedigree of the proband SMA patient. *SMN1/2* copy number is indicated per individual. Markers lengths per haplotype are shown as numbers in the colored boxes. Reported markers from top to bottom: D5S465, D5S681, D5S629, D5S1417, D5S1408, D5S610, D5S637, D5S1501.

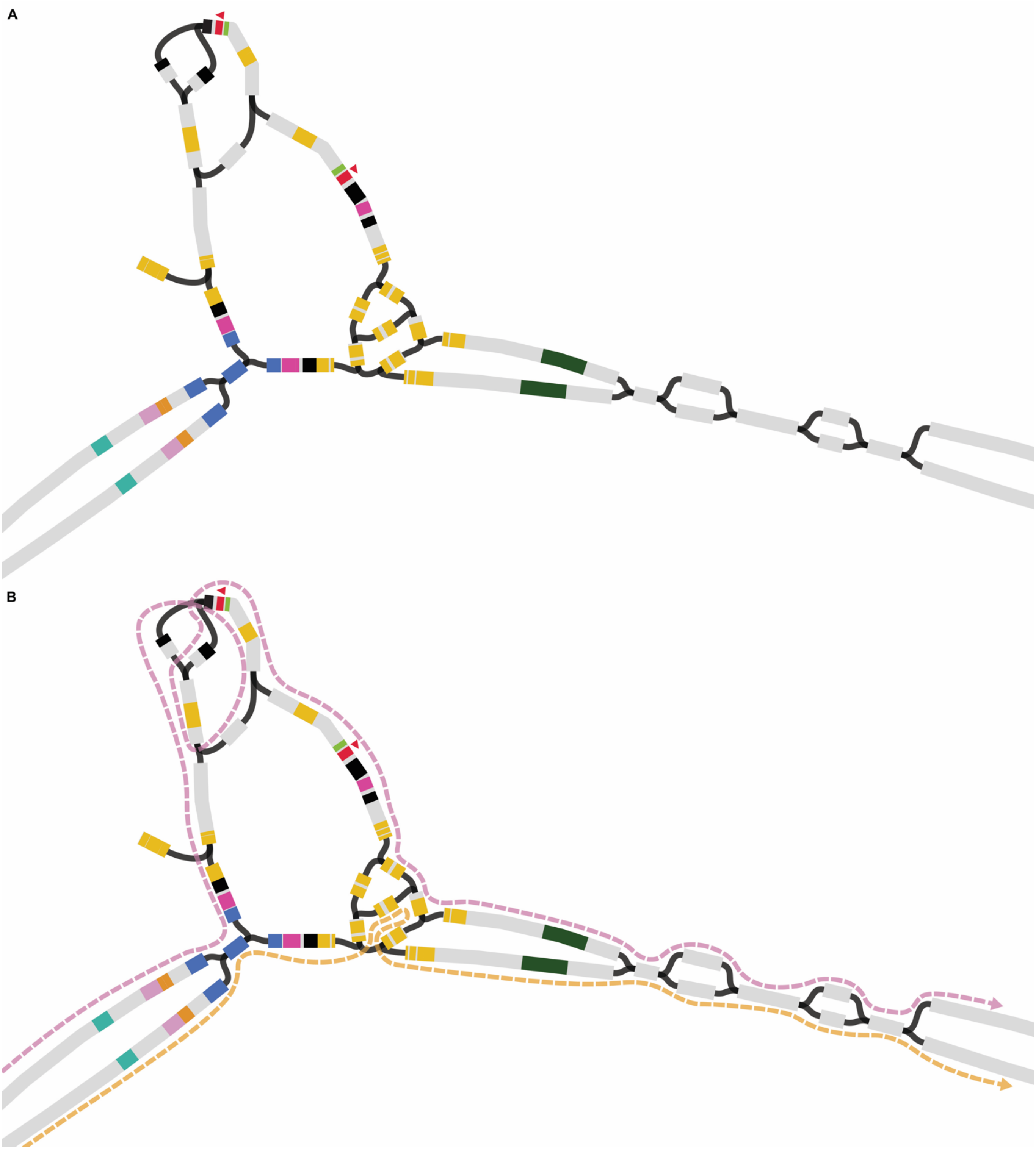

Supplementary figure 2: Unphased assembly graph of proband.
(A) Unphased assembly graph of the proband, visualized with Bandage. Contigs are represented by gray boxes, edges by black lines. Genes are indicated by the same colors as in **Fig. 3A**. *SMN1/2* genes are indicated by red arrows.
(B) Likely paths through the assembly to form the alleles of the proband as shown in **Fig. 3A**. The path along the paternal allele is indicated in purple, the path along the maternal allele in yellow.

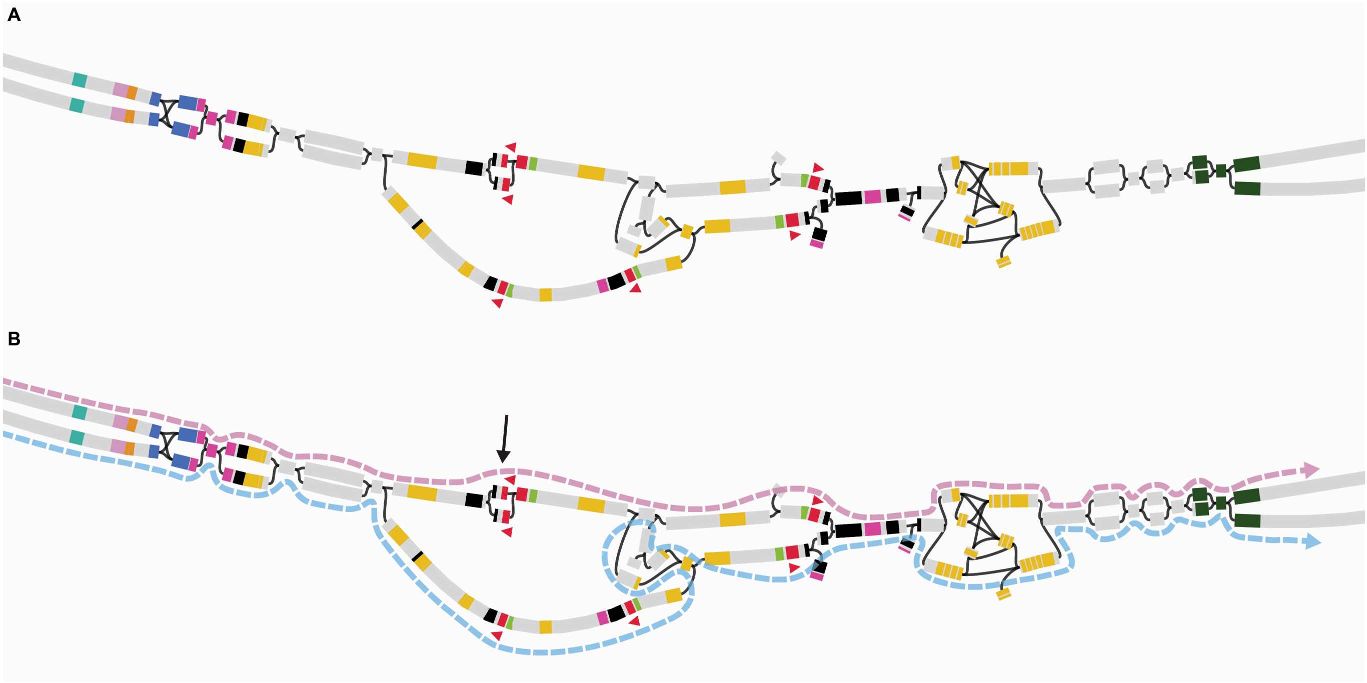

Supplementary figure 3: Unphased assembly graph of father.
(A) Unphased assembly graph of the father, visualized with Bandage. Contigs are represented by gray boxes, edges by black lines. Genes are indicated by the same colors as in **Fig. 3A**. *SMN1/2* genes are indicated by red arrows.
(B) Likely paths through the assembly to form the alleles of the father. The purple path likely contains a duplication that was collapsed in the assembly (indicated with a black arrow), and therefore only contains two instead of three *SMN* copies.

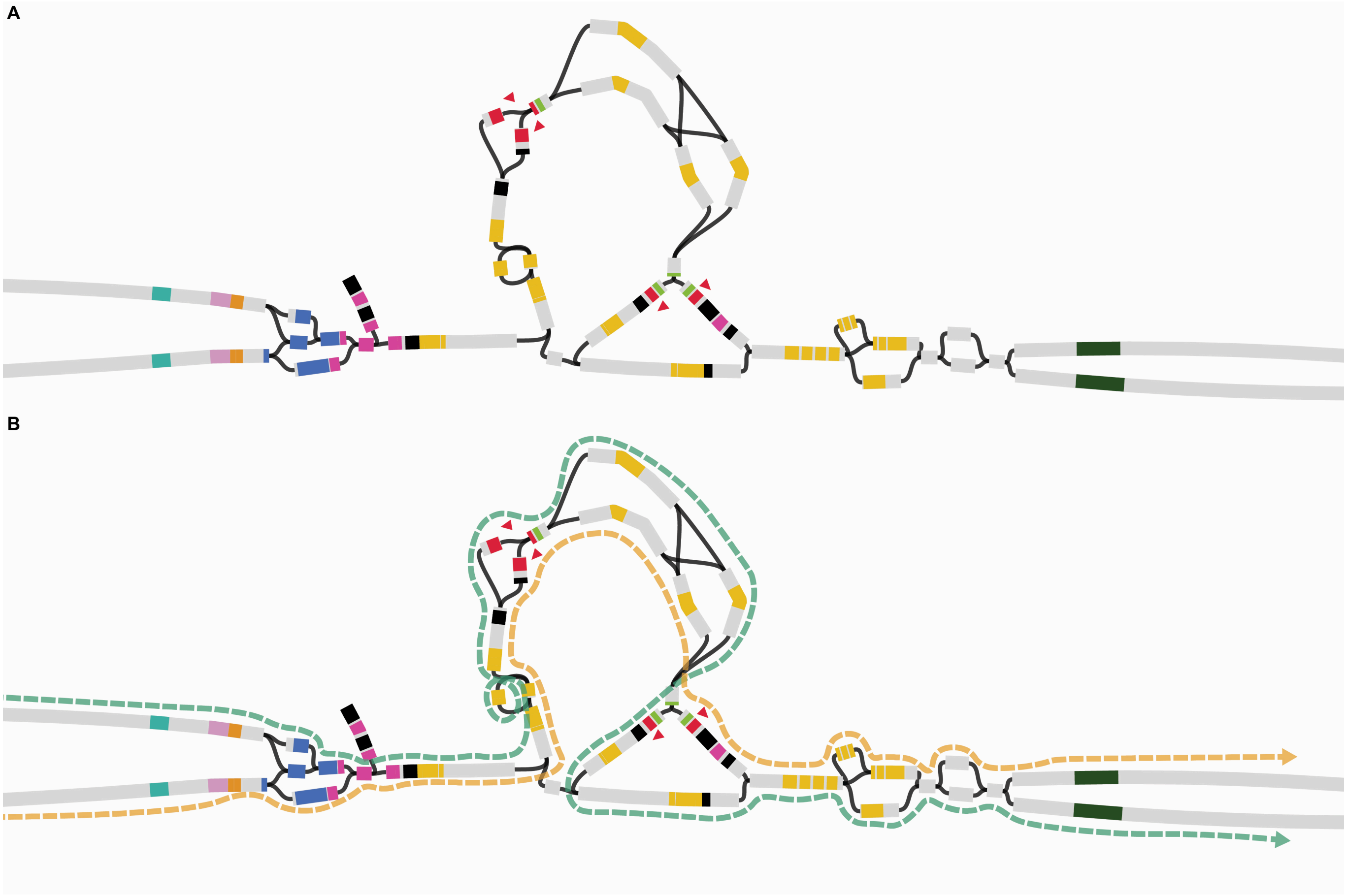

Supplementary figure 4: Unphased assembly graph of mother.
(A) Unphased assembly graph of the mother, visualized with Bandage. Contigs are represented by gray boxes, edges by black lines. Genes are indicated by the same colors as in **Fig. 3A**. *SMN1/2* genes are indicated by red arrows.
(B) Likely paths through the assembly to form the alleles of the mother.

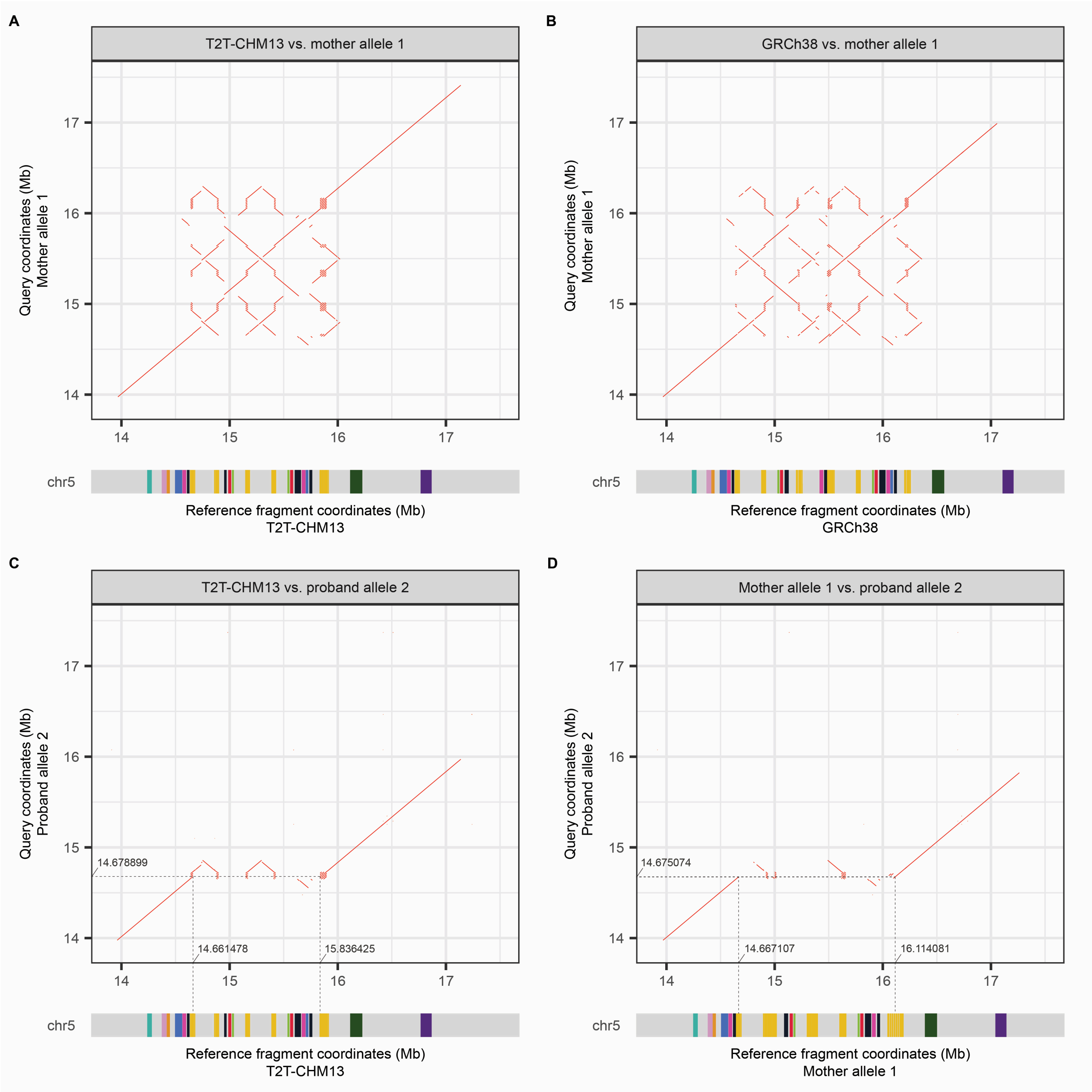

Supplementary figure 5: Dot plots comparing different resolved alleles to reference genomes and to each other.
The shown coordinates correspond to the assembled fragment of 30Mb, roughly corresponding to GRCh38 chr5:55,000,000-85,000,000.
(A) Dot plot of allele 1 of the mother (y-axis) against the T2T-CHM13 reference genome (x-axis): the *SMN* genes are in the same orientation.
(B) Dot plot of allele 1 of the mother (y-axis) against the GRCh38 reference genome (x-axis): the *SMN2* gene is in opposite orientation. This allele is more similar to T2T-CHM13 (A) than GRCh38, and contains more repetitive sequences (short diagonal lines above each other) than in both reference genomes.
(C) Dot plot of (maternal) allele 2 of the proband (y-axis) against the T2T-CHM13 reference genome (x-axis): the deletion of the sequence containing *SMN1* and *SMN2* is visible as a gap in the diagonal line. The exact length of the

deletion relative to T2T-CHM13 as determined from the nucmer output is 1174947bp (coordinates 14661478-15836425 on aligned fragment, translating to chr5:70483959-71658906 on T2T-CHM13).

(D) Dot plot of (maternal) allele 2 of the proband (y-axis) against allele 1 of the mother (x-axis): the deletion of the sequence containing *SMN1* and *SMN2* is visible as a gap in the diagonal line. The exact length of the deletion relative to allele 1 of the mother as determined from the nucmer output is 1446974bp (coordinates 14667107-16114081 on aligned fragment).

Supplementary table 1: Sanger sequencing results used for determining the inheritance pattern of *SMN1/2* copies in **Fig. 2**.

INDIVIDUAL	SNV 3	SNV 4	SNV 5	SNV 7	SNV 9	SNV 10	SNV 12	SNV 13
**I-1**	C	C	C/G	A	G/A	C	A/G	G
**I-2**	C/T	C/T	C	A	G	C	A	G/A
**I-3**	C	C	C	A/G	G	C	A	G/A
**I-5**	C	C	C	A	G	C/T	A	G/A
**I-6**	C	C/T	C	A	G	C	A	G/A
**II-1**	C/T	C/T	C/G	A	G/A	C	A/G	G/A
**II-2**	C	C	C	A/G	G	C/T	A	G/A
**II-3**	C	C	C	A	G	C	A	G/A
**II-4**	C	C	C	A/G	G	C/T	A	G/A
**II-5**	C	C/T	C	A	G	C	A	G
**II-6**	C	C	C	A	G	C	A	G/A
**III-1**	C	C	C/G	A	G/A	C	A/G	G
